# Cause-Specific Mortality in HIV-Positive Patients Who Survived Ten Years after Starting Antiretroviral Therapy

**DOI:** 10.1371/journal.pone.0160460

**Published:** 2016-08-15

**Authors:** Adam Trickey, Margaret T. May, Janne Vehreschild, Niels Obel, Michael John Gill, Heidi Crane, Christoph Boesecke, Hasina Samji, Sophie Grabar, Charles Cazanave, Matthias Cavassini, Leah Shepherd, Antonella d’Arminio Monforte, Colette Smit, Michael Saag, Fiona Lampe, Vicky Hernando, Marta Montero, Robert Zangerle, Amy C. Justice, Timothy Sterling, Jose Miro, Suzanne Ingle, Jonathan A. C. Sterne, Antiretroviral Therapy Cohort Collaboration (ART-CC)

**Affiliations:** 1 School of Social and Community Medicine, University of Bristol, Bristol, United Kingdom; 2 German Centre for Infection Research, partner site Bonn-Cologne, Cologne, Germany; 3 Department I for Internal Medicine, University Hospital of Cologne, Cologne, Germany; 4 Department of Infectious Diseases, Copenhagen University Hospital, Copenhagen, Denmark; 5 Division of Infectious Diseases, University of Calgary, Calgary, Canada; 6 Center for AIDS Research, University of Washington, Seattle, WA, United States of America; 7 Department of Internal Medicine, University of Bonn, Bonn, Germany; 8 Epidemiology and Population Health Program, British Columbia Centre for Excellence in HIV/AIDS, Vancouver, Canada, and Faculty of Health Sciences, Simon Fraser University, Burnaby, Canada; 9 Sorbonne Universités, UPMC Univ Paris 06, UMR_S 1136, Institut Pierre Louis d’Epidémiologie et de Santé Publique, F-75013, Paris, France; 10 INSERM, UMR_S 1136, Institut Pierre Louis d’Epidémiologie et de Santé Publique, F-75013, Paris, France; 11 Université Paris Descartes et Assistance Publique-Hôpitaux de Paris, Groupe hospitalier Cochin Hôtel-Dieu, Paris, France; 12 Centre Hospitalier Universitaire de Bordeaux, Hôpital Pellegrin, Bordeaux, F-33000, France; 13 Service of Infectious Diseases, Lausanne University Hospital and University of Lausanne, Lausanne, Switzerland; 14 Research Department of Infection and Population Health, UCL Medical School, London, United Kingdom; 15 Clinic of Infectious Diseases & Tropical Medicine, San Paolo Hospital, University of Milan, Milan, Italy; 16 Stichting HIV Monitoring, Amsterdam, the Netherlands; 17 Division of Infectious Disease, Department of Medicine, University of Alabama, Birmingham, United States of America; 18 Red de Investigación en Sida, Centro Nacional de Epidemiología, Instituto de Salud Carlos III, Avda. Monforte de Lemos, 5 28029, Madrid, Spain; 19 La Fe Hospital, Valencia, Spain; 20 Innsbruck Medical University, Innsbruck, Austria; 21 Yale University School of Medicine, New Haven, CT, United States of America, and VA Connecticut Healthcare System, West Haven, CT, United States of America; 22 Vanderbilt University School of Medicine, Nashville, TN, United States of America; 23 Hospital Clínic-IDIBAPS, University of Barcelona, Barcelona, Spain; University Hospital Zurich, SWITZERLAND

## Abstract

**Objectives:**

To estimate mortality rates and prognostic factors in HIV-positive patients who started combination antiretroviral therapy between 1996–1999 and survived for more than ten years.

**Methods:**

We used data from 18 European and North American HIV cohort studies contributing to the Antiretroviral Therapy Cohort Collaboration. We followed up patients from ten years after start of combination antiretroviral therapy. We estimated overall and cause-specific mortality rate ratios for age, sex, transmission through injection drug use, AIDS, CD4 count and HIV-1 RNA.

**Results:**

During 50,593 person years 656/13,011 (5%) patients died. Older age, male sex, injecting drug use transmission, AIDS, and low CD4 count and detectable viral replication ten years after starting combination antiretroviral therapy were associated with higher subsequent mortality. CD4 count at ART start did not predict mortality in models adjusted for patient characteristics ten years after start of antiretroviral therapy. The most frequent causes of death (among 340 classified) were non-AIDS cancer, AIDS, cardiovascular, and liver-related disease. Older age was strongly associated with cardiovascular mortality, injecting drug use transmission with non-AIDS infection and liver-related mortality, and low CD4 and detectable viral replication ten years after starting antiretroviral therapy with AIDS mortality. Five-year mortality risk was <5% in 60% of all patients, and in 30% of those aged over 60 years.

**Conclusions:**

Viral replication, lower CD4 count, prior AIDS, and transmission via injecting drug use continue to predict higher all-cause and AIDS-related mortality in patients treated with combination antiretroviral therapy for over a decade. Deaths from AIDS and non-AIDS infection are less frequent than deaths from other non-AIDS causes.

## Introduction

HIV-positive patients who started combination antiretroviral therapy (ART) soon after it became widely available in Europe and North America in 1996 have been treated for up to 20 years[1]. Current mortality rates in these patients are of great interest: they started ART regimens that are less tolerable and have lower antiviral potency than those now available[2] and are likely to have switched ART regimen repeatedly as better drugs became available[3, 4]. As they age these patients are at increased risk of age-related comorbidities such as cardiovascular disease, cancer, liver and renal disease[5, 6]. The proportion of deaths classified as AIDS has decreased over this period[7, 8]. Prognosis beyond the first decade of ART is thus of interest to patients, their treating physicians, mathematical modellers, and those planning health resources.

In the early ART era, mortality was high in the first year of ART and decreased thereafter[9, 10]. For patients who started treatment with immune suppression, AIDS-related causes of death dominate soon after starting ART, but later decline relative to non-AIDS causes[5, 11, 12]. For some successfully treated patient groups mortality rates may be similar to those of the background population[13, 14].

Several factors predict short-term mortality in HIV positive patients starting ART[9, 10], but the prognostic value of some of these factors (for example CD4 count at ART start) diminishes with time[15]. We studied factors that are prognostic for mortality in a large patient cohort who started combination ART without previous exposure to antiretroviral drugs and were treated for a decade. We determined all-cause and cause-specific mortality rates and identified clinical and demographic risk factors for all-cause and cause-specific mortality present ten years after starting ART.

## Methods

The Antiretroviral Therapy Cohort Collaboration (ART-CC) combined data from HIV cohorts in North America and Europe on HIV-1 positive patients aged at least 16 years who started ART with at least three drugs[16] without prior exposure to antiretroviral medications. Further details on the dataset can be found elsewhere (www.art-cohort-collaboration.org). Cohorts were approved by ethics committees or institutional review boards, used standardized methods of data collection, and scheduled follow-up visits at least every six months. Data were analysed anonymously. Eligible patients started ART during 1996–9, remained alive and in follow-up for at least ten years after ART start, and had at least one CD4 count and viral load measurement between nine and ten years after ART start. We used the CD4 count measured before and closest to ART start date plus 10 years. Patients who had stopped or interrupted ART were included. Data analysed were compiled from 18 cohorts (Appendix 1) with follow up until 31st July 2013.

Information relevant to assigning causes of death was obtained either through linkage with Vital Statistics agencies and hospitals or through physician report and active follow-up. We adapted the Cause of Death (CoDe) project protocol[17] (www.cphiv.dk/CoDe.aspx) to classify causes of death. If ICD-10 codes were available, causes of death were classified by a clinician and a computer algorithm[18]. When ICD-10 codes were not available, two clinicians independently classified each death. Disagreements between clinicians and/or computer-assigned codes were resolved via panel discussion[5]. Deaths were coded as AIDS-related if there was a serious AIDS defining condition prior to death and/or a low CD4 count (<100/μL) within a year (18 months if off treatment) of death, and a diagnosis compatible with AIDS as cause of death[6]. All other deaths, including those of unknown cause, were considered non-AIDS related. Cause of death information was analysed for six cohorts for which causes had been classified for over 70% of deaths.

### Statistical Methods

Follow-up started ten years after ART start and ended at the earliest of death, loss to follow-up (LTFU) or administrative censoring (cohort-specific database close date). Patients were considered LTFU at their last clinical observation if this was over a year before the close date. AIDS was categorised as: no recorded AIDS diagnosis; first AIDS diagnosis before starting ART; and first AIDS diagnosis between starting ART and start of follow-up. Drug regimens were derived at ART start and start of follow-up and were classified as NNRTI-based, PI-based, other regimen, or no drugs at start of follow-up.

We compared characteristics of eligible patients with those no longer followed-up at ten years after start of ART because they died, were LTFU or transferred to another treatment centre. We estimated unadjusted mortality rates assuming constant rates during follow-up. Five-year cumulative mortality was estimated using the Kaplan-Meier method. We used Cox models to estimate unadjusted and adjusted hazard ratios (HR): adjustments were for sex, regimen, age (16–39, 40–49, 50–59, 60–69, ≥70 years), mode of transmission (injection drug use [IDU] or not), and HIV-1 RNA (0–50, 51–1000, ≥1001copies/mL), CD4 count (0–99, 100–199, 200–349, 350–499, 500–749, ≥750 cells/μL) and AIDS status at start of follow-up, chosen due to prior literature[19]. We investigated whether CD4 count at ART start was associated with mortality after accounting for the association of CD4 count 10 years after ART start.

We tabulated frequencies of AIDS, non-AIDS, and specific causes of death. We estimated adjusted HR for the more frequent (n≥10) specific causes of death (AIDS, cardiovascular, liver-related, malignancies (non-AIDS and not hepatitis-related), suicide/accidents) stratified by age (≥60 vs. <60 years), sex, IDU status, CD4 count (<200 vs. ≥200 cells/μL), viral suppression (HIV-1 RNA >50 vs. ≤50 copies/mL), and AIDS diagnosis during or before the first decade of ART (vs. no AIDS). Five-year cumulative risks of death, according to age, risk group and CD4 count, viral load and AIDS status recorded ten years after starting ART, were estimated using cumulative incidence functions from a flexible Weibull model[20], stratified by IDU and CD4 count. We estimated five-year mortality risk for each patient and tabulated the frequency distribution of patients grouped by five-year mortality risk (0–1.99, 2–4.99, 5–9.99, 10–24.99 and ≥25%) by age (<60 and ≥60 years). We compared five-year mortality risk in HIV-positive individuals with that of age-matched French general population in 2013 using mortality.org data. We chose the French population as comparator because the largest proportion of patients were treated in France. As a sensitivity analysis we used competing risks regression with LTFU and death as the competing outcomes[21]. Analyses were done using Stata 13 (StataCorp, Texas, USA).

## Results

24,445 patients started ART between 1996–1999. Of these, 3,577 (15%) died, 5,963 (24%) were LTFU during the first decade of ART, and 1,894 (8%) were excluded because CD4 count or viral load were not available at start of follow-up leaving 13,011 (53%) patients eligible for analyses. Table 1 compares characteristics at ART start of eligible patients with excluded patients who died, were LTFU, or lacked a measurement of CD4 and viral load between nine and ten years after ART start. The two groups had similar age distributions, but eligible patients were less likely to be IDU (1,636 [13%] vs 2,623 [23%]), had higher CD4 count and lower viral load at ART start.

**Table 1:** Characteristics (at start of ART) of patients who were and were not eligible for analyses of prognosis from 10 years after start of ART.

	Patients who started ART 1996–1999 but were excluded from analyses		Patients included in analyses of prognosis from 10 years after start of ART
Characteristics	Number (%) of patients	Number (%) of deaths	Numbers (%) of patients
**Total**	11,434 (100%)		13,011 (100%)
**IDU**	2,623 (23%)	820 (31%)	1,636 (13%)
**Female**	2,215 (19%)	472 (21%)	2,701 (21%)
**AIDS diagnosis**	2,613 (23%)	1,250 (48%)	2,714 (21%)
Age (years)			
Median (IQR)	36 (31, 43)		36 (31,43)
16–29	2,127 (19%)	330 (16%)	2,330 (18%)
30–39	5,147 (45%)	1,335 (26%)	6,029 (46%)
40–49	2,600 (23%)	1,148 (44%)	3,129 (24%)
50–59	1,089 (10%)	614 (56%)	1,181 (9%)
≥60	471 (4%)	296 (63%)	342 (3%)
CD4 count (cell/μL)			
Median (IQR)	224 (85, 335)		250 (100, 401)
≥750	381 (3%)	70 (19%)	423 (3%)
500–749	1,166 (10%)	251 (22%)	1,483 (11%)
350–499	1,868 (16%)	408 (22%)	2,390 (18%)
200–349	2,799 (24%)	776 (28%)	3,372 (26%)
100–199	2,092 (18%)	775 (37%)	2,120 (16%)
50–99	1,103 (10%)	469 (43%)	1,229 (9%)
25–49	760 (7%)	340 (45%)	748 (6%)
0–24	1,265 (11%)	634 (50%)	1,246 (10%)
HIV 1 RNA cell count			
Median (IQR)	7.4×10^4^ (1.7×10^4^, 2.4×10^5^)		7.1×10^4^ (1.7×10^4^, 2.3×10^5^)
0–50	104 (1%)	11 (11%)	88 (1%)
51–1000	580 (5%)	106 (18%)	669 (5%)
1001–100000	5,766 (50%)	1,645 (29%)	6,794 (52%)
>100000	4,984 (44%)	1,961 (39%)	5,460 (42%)

LTFU: lost to follow-up; IDU: injection drug use; IQR: inter-quartile range

Among 13,011 patients during follow-up from 10 years after starting ART, there were 656 deaths during 50,593 person years giving a crude mortality rate of 12.9 [95% CI 12.0,14.0] per 1,000 person years, and estimated five year survival 93.5% (93.1–94.2%). Median (IQR) follow-up was 4.0 (3.1–5.0) years and maximum follow-up time varied between cohorts from 4.5 to 7.1 years. The rate of LTFU was 5.5 (95% CI 5.3–5.7) per 100 person years (N = 5,963). Patients LTFU after 10 years were more likely to be female, IDU and younger (Table 2). Median (IQR) CD4 count at ten years after starting ART was lower in those who died, but was similar in those LTFU or who remained in care: 383 (185–554), 535 (350–740), and 566 (390–774) cells/μL, respectively. The proportions of patients with HIV-RNA ≤50 copies/mL at ten years after starting ART was lower in those who died, but similar in those LTFU or who remained in care: 56%, 73%, and 77%, respectively (Table 2).

**Table 2:** Characteristics of patients followed from 10 years after start of ART who died, were lost to follow up (LTFU) or remained in the study until end of follow up.

	Number (%) of patients		
	Died (N = 656)	LTFU (N = 2,708)	Remained in the study (N = 9,647)
IDU	121 (18%)	474 (18%)	1,041 (11%)
Female	81 (12%)	669 (25%)	1,951 (20%)
No AIDS	302 (46%)	1,867 (69%)	6,492 (67%)
AIDS before ART start	175 (27%)	537 (20%)	2,002 (21%)
AIDS between ART start and 10 years afterwards	179 (27%)	304 (11%)	1,153 (12%)
Age (years)			
Median (IQR)	52 (45, 60)	45 (40, 51)	46 (41, 53)
16–39	53 (8%)	606 (22%)	1,671 (17%)
40–49	225 (34%)	1,287 (48%)	4,517 (47%)
50–59	200 (30%)	570 (21%)	2,359 (24%)
≥60	178 (27%)	245 (9%)	1,100 (11%)
CD4 count (cells/μL)			
Median (IQR)	383 (185, 554)	535 (350, 740)	566 (390, 774)
0–99	98 (15%)	97 (4%)	205 (2%)
100–199	81 (12%)	176 (7%)	371 (4%)
200–349	131 (20%)	398 (15%)	1,321 (14%)
350–499	128 (20%)	532 (20%)	1,984 (21%)
500–749	126 (19%)	856 (32%)	3,111 (32%)
≥750	92 (14%)	649 (24%)	2655 (28%)
Viral load (HIV-1 RNA copies/mL)			
% with RNA≤50	56%	73%	77%
0–50	370 (56%)	1,977 (73%)	7,467 (77%)
51–1000	119 (18%)	365 (13%)	1,206 (13%)
>1000	167 (25%)	366 (14%)	974 (10%)

IDU: injection drug use; IQR: inter-quartile range

Patient demographics and clinical characteristics at start of follow-up (ten years after starting ART) are shown in Table 3. Median (IQR) CD4 count was 250 (100–401) cells/μL at ART start and increased to 550 (371–760) cells/μL at ten years. The correlation between CD4 count at ART start and at ten years was 0.27 (p<0.001). Most patients (11,504; 88%) had HIV-1 RNA ≤200 copies/mL ten years after ART start. At ART start most patients (10,266; 79%) were on PI-based regimens with 2,229 (17%) on NNRTI-based regimens. By ten years 4,555 (35%) and 5,002 (39%) of patients were on PI- and NNRTI-based regimens, respectively, whilst 1,072 (8%) were off ART. Of the remaining patients, 1,853 (14%) were only receiving NRTIs, 108 (1%) and 9 (0.1%) were on CCR5 receptor antagonist- and integrase inhibitor-based regimens respectively and 3% were on other regimens. Of 400 (3%) patients with CD4 count <100 cells/μL ten years after ART start, 71 (18%) were not on treatment, 1,001 (8%) of those with CD4 count ≥100 cells/μL ten years after ART start were not on treatment. The proportions of virally suppressed patients (HIV-1 RNA <50 copies/mL) were 30% and 77% for those with CD4 counts <100 and ≥100 cells/μL, respectively. There were 6,287 (48%) patients with HIV-1 RNA <50 copies/mL and with CD4 count ≥500 cells/μL.

**Table 3:** Characteristics of eligible patients 10 years after start of ART, together with unadjusted and adjusted hazard ratios (HR).

Characteristics	Number (%) of patients	Number (%) of deaths	Unadjusted HR	Adjusted HR
			(95% CI)	(95% CI)*
**Total**	13,011 (100%)	656 (100%)		
**Risk group**				
IDU (vs non-IDU)	1,636 (13%)	121 (7%)	2.54 (2.03, 3.18)	2.58 (2.04, 3.26)
**Sex**				
Female (vs male)	2,701 (21%)	81 (3%)	0.76 (0.60, 0.96)	0.95 (0.74, 1.21)
**AIDS diagnosis**				
No AIDS	8,661 (67%)	302 (3%)	1	1
AIDS before ART start	2,714 (21%)	175 (6%)	1.73 (1.43, 2.09)	1.48 (1.23, 1.80)
AIDS between ART start and 10 years afterwards	1,636 (13%)	179 (11%)	2.14 (1.75, 2.62)	1.68 (1.37, 2.06)
**Age (years)**				
Median (IQR)	46 (41, 53)			
16–39	2,330 (18%)	53 (2%)	1	1
40–49	6,029 (46%)	225 (4%)	1.50 (1.11, 2.03)	1.40 (1.04, 1.90)
50–59	3,129 (24%)	200 (6%)	2.15 (1.58, 2.92)	2.14 (1.56, 2.92)
60–69	1,181 (9%)	123 (10%)	3.39 (2.44, 4.71)	4.29 (3.06, 6.02)
≥70	342 (3%)	55 (16%)	6.18 (4.22, 9.04)	8.59 (5.82, 12.7)
**CD4 count (cells/μL)**				
Median (IQR)	550 (372, 760)			
≥750	3,396 (26%)	92 (3%)	1	1
500–749	4,093 (31%)	126 (3%)	1.12 (0.85, 1.46)	1.01 (0.78, 1.33)
350–499	2,644 (20%)	128 (5%)	1.72 (1.32, 2.25)	1.41 (1.07, 1.84)
200–349	1,850 (14%)	131 (7%)	2.37 (1.81, 3.10)	1.80 (1.37, 2.37)
100–199	628 (5%)	81 (13%)	4.28 (3.16, 5.78)	3.07 (2.24, 4.19)
0–99	400 (3%)	98 (25%)	9.25 (6.90, 12.4)	6.17 (4.46, 8.53)
**Viral load (HIV-1 RNA copies/mL)**				
Median (% with RNA≤50)	50 (75%)			
0–50	9,814 (75%)	370 (4%)	1	1
51–1000	1,690 (13%)	119 (7%)	1.25 (1.00, 1.56)	1.09 (0.87, 1.36)
>1000	1,507 (12%)	167 (11%)	2.41 (1.99, 2.91)	1.74 (1.40, 2.15)

IDU: injection drug use; IQR: inter-quartile range; CI: confidence interval

*Models adjusted for all variables in table, stratified by cohort.

### Mortality from ten years after start of ART

Mortality was substantially higher in patients aged 60–69 and >70 years from ten years after starting ART (adjusted HR 4.29 [95% CI 3.06–6.02] and 8.59 [5.82–12.7] respectively compared with age 16–39 (Table 3)). Mortality was also substantially higher in patients with CD4 count <100 cells/μL from ten years after ART start (adjusted HR 6.17 [4.46–8.53] compared with ≥750 cells/μL). Mortality rates were similar in patients with CD4 count 500–749 and ≥750 cells/μL. Mortality was higher in IDU compared with non-IDU (adjusted HR 2.58 [2.04–3.26]). Viral load >1000 copies/mL at ten years and an AIDS diagnosis in the first ten years after starting ART were associated with higher subsequent mortality (adjusted HR 1.74 [1.40–2.15] and 1.68 [1.37–2.06] respectively). There was no difference in subsequent mortality between the patients on PI- (adjusted HR 1.17 [0.95–1.43] compared with NNRTI-) and NNRTI-based regimens at 10 years after ART start.

Lower CD4 count at ART start was weakly associated with higher mortality from ten years (Table 4). This was attenuated by adjustment for CD4 count at ten years after starting ART and other covariates, with a suggestion of lower subsequent mortality for patients with very low CD4 count at ART start after adjustment for ten year CD4 count [HR = 0.62 (0.46–0.83) for CD4 <50 vs. ≥350 cells/μL].

**Table 4:** Unadjusted and adjusted hazard ratios (HR) according to CD4 count at start of ART and at 10 years after start of ART.

	Cells/μL	N (%)	Unadjusted HR (95% CI)	Adjusted HR (95% CI)*
**CD4 count at start of ART (cells/μL)**	≥350	4,296 (33%)	1	1
	200–349	3,372 (26%)	1.09 (0.88, 1.35)	0.96 (0.77, 1.19)
	100–199	2,120 (16%)	1.38 (1.09, 1.73)	0.94 (0.74, 1.20)
	50–99	1,229 (9%)	1.60 (1.24, 2.08)	0.88 (0.67, 1.16)
	0–49	1,994 (15%)	1.19 (0.94, 1.52)	0.69 (0.52, 0.90)
**CD4 10 years after start of ART (cells/μL)**	≥750	3,396 (26%)	1	1
	500–749	4,093 (31%)	1.11 (0.85, 1.46)	1.04 (0.80, 1.37)
	350–499	2,644 (20%)	1.72 (1.32, 2.25)	1.47 (1.12, 1.93)
	200–349	1,850 (14%)	2.37 (1.81, 3.10)	1.92 (1.45, 2.54)
	100–199	628 (5%)	4.28 (3.16, 5.78)	3.33 (2.42, 4.60)
	0–99	400 (3%)	9.25 (6.90, 12.40)	6.85 (4.89, 9.60)

CI: Confidence interval

*Adjusted for sex, regimen, age (16–39, 40–49, 50–59, 60–69, ≥70 years), mode of transmission (IDU or not), HIV-1 RNA levels 10 years after start of ART (0–50, 51–1000, >1000 copies/ml), AIDS status and mutually adjusted for both CD4 measurements and stratified by cohort.

Results were similar to those in the main analysis when LTFU was considered a competing risk to death in the sensitivity analysis.

### Five-year mortality risk

**Fig 1:**
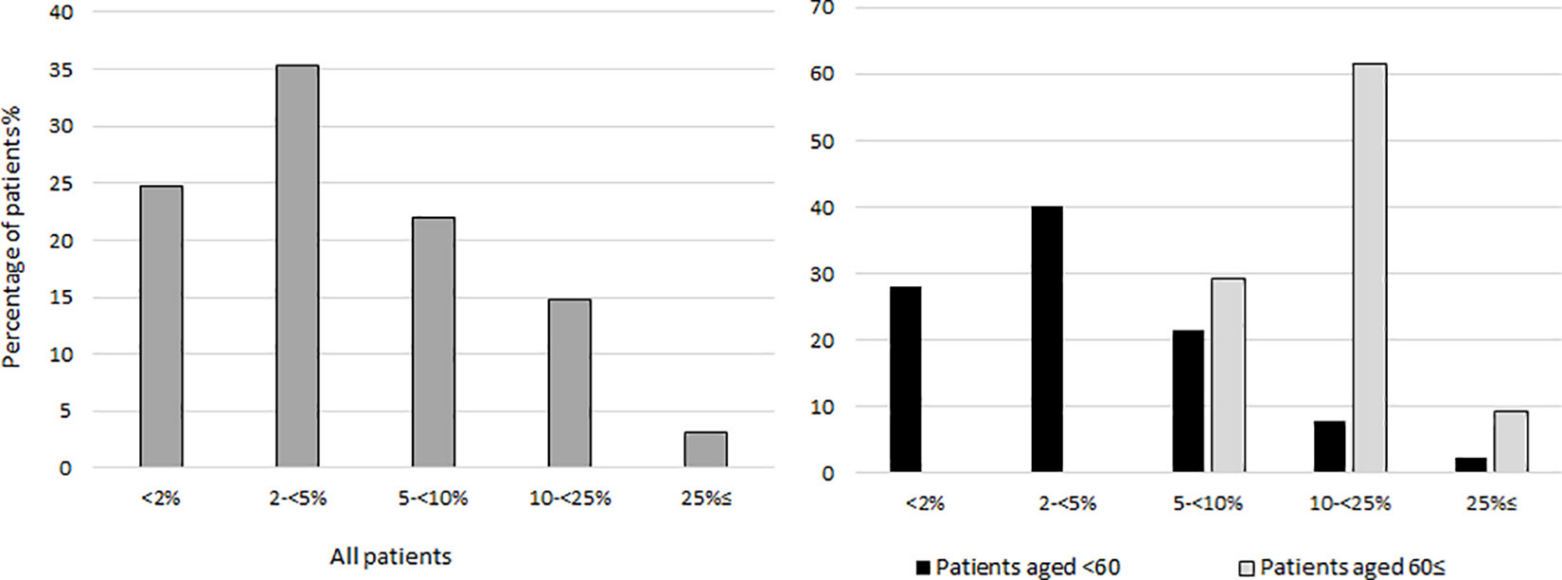
Frequency distribution of patients grouped by five-year mortality risk, overall and by age-group.

Table 5 shows estimated five-year mortality risk from ten years after ART start in groups defined by combinations of age, IDU risk group, AIDS status, CD4 count and viral suppression. For patients aged 40–49 years at ten years after starting ART the subsequent five-year mortality risk ranged from 1.9% (1.5–2.4%) in those who were non-IDU and virally suppressed with CD4 count >500 cells/μL and no AIDS, to 48% (40–58%) in those who were IDU and not virally suppressed with CD4 count <100 cells/μL and AIDS during the first decade of ART. In comparison, the 5-year mortality risk in the French general population of the same age was 1%. Fig 1 shows the proportion of patients in five-year mortality risk strata, for younger (aged <60 years) and older (≥60) patients. Five-year mortality risk was below 25% in 12,649 (97%) patients, below 10% in 10,715 (82%) patients and below 5% in 7,829 (60%). Mortality risk was strongly age-related: only 384 (29%) patients aged ≥60 years had five-year mortality risk less than 10%.

**Table 5:** 5-year percentage risk of death (95% CI) from 10 years after start of ART, according to age, IDU risk group, AIDS status, CD4 count and viral suppression (HIV RNA <50 copies/mL)^*^.

	CD4 count 10 years after start of ART (cells/μL)					
	0–99	100–199	200–349	350–499	500–749	>750
**Non-IDU, No AIDS, Virologically suppressed**						
Age 16–39	6.9 (4.9–9.8)	3.9 (2.7–5.5)	2.2 (1.6–3)	1.7 (1.2–2.3)	1.2 (0.9–1.6)	1.2 (0.8–1.6)
Age 40–49	10.9 (8.2–14.3)	6.1 (4.6–8.1)	3.5 (2.7–4.4)	2.7 (2.1–3.4)	1.9 (1.5–2.4)	1.9 (1.5–2.4)
Age 50–59	18.8 (14.3–24.4)	10.8 (8.2–14.2)	6.1 (4.8–7.8)	4.8 (3.8–6)	3.4 (2.7–4.3)	3.3 (2.6–4.3)
Age 60+	36.4 (28.2–46.2)	22.1 (16.9–28.5)	12.9 (10.3–16.2)	10.1 (8.1–12.7)	7.2 (5.7–9.1)	7.1 (5.5–9.1)
**Non-IDU, No AIDS, Not virologically suppressed**						
Age 16–39	11.3 (8.2–15.4)	6.4 (4.6–8.8)	3.6 (2.6–4.9)	2.8 (2–3.8)	2 (1.4–2.7)	1.9 (1.4–2.7)
Age 40–49	17.5 (13.7–22.1)	10 (7.7–13.1)	5.7 (4.5–7.2)	4.4 (3.5–5.6)	3.1 (2.4–4)	3.1 (2.4–4)
Age 50–59	29.3 (23.3–36.4)	17.4 (13.4–22.3)	10 (7.9–12.6)	7.8 (6.1–9.9)	5.6 (4.4–7.1)	5.5 (4.2–7.2)
Age 60+	53 (43.1–63.6)	34 (26.6–42.8)	20.6 (16.4–25.7)	16.3 (12.8–20.6)	11.8 (9.1–15.1)	11.6 (8.8–15.2)
**Non-IDU, AIDS, Virologically suppressed**						
Age 16–39	11.3 (8.1–15.8)	6.4 (4.6–9)	3.6 (2.6–4.9)	2.8 (2–3.8)	2 (1.4–2.7)	1.9 (1.4–2.7)
Age 40–49	17.6 (13.7–22.4)	10.1 (7.7–13.1)	5.7 (4.5–7.2)	4.4 (3.5–5.6)	3.2 (2.5–4)	3.1 (2.4–4)
Age 50–59	29.4 (23.3–36.8)	17.5 (13.6–22.3)	10.1 (8.1–12.6)	7.9 (6.3–9.8)	5.6 (4.5–7)	5.5 (4.3–7.1)
Age 60+	53.2 (43.4–63.8)	34.2 (27.1–42.6)	20.7 (16.8–25.5)	16.4 (13.2–20.2)	11.8 (9.5–14.8)	11.7 (9.1–14.9)
**Non-IDU, AIDS, Not virologically suppressed**						
Age 16–39	18.2 (13.4–24.3)	10.5 (7.6–14.4)	5.9 (4.3–8.1)	4.6 (3.3–6.3)	3.3 (2.4–4.5)	3.2 (2.3–4.6)
Age 40–49	27.6 (22.3–33.7)	16.3 (12.7–20.7)	9.4 (7.4–11.8)	7.3 (5.7–9.3)	5.2 (4–6.7)	5.1 (3.9–6.8)
Age 50–59	44.1 (36.7–52.2)	27.4 (21.9–33.9)	16.3 (13.1–20.1)	12.8 (10.2–16)	9.2 (7.2–11.7)	9 (6.9–11.8)
Age 60+	71.8 (62.1–80.9)	50.2 (41–60.2)	32.1 (26.1–39.1)	25.8 (20.7–31.9)	19 (14.9–24)	18.7 (14.2–24.3)
**IDU, No AIDS, Virologically suppressed**						
Age 16–39	13.5 (9.5–19.2)	7.7 (5.4–11)	4.3 (3.1–6.1)	3.4 (2.4–4.7)	2.4 (1.7–3.4)	2.3 (1.6–3.3)
Age 40–49	20.9 (15.8–27.2)	12.1 (9–16.1)	6.9 (5.3–8.9)	5.4 (4.1–7)	3.8 (2.9–5)	3.7 (2.8–5)
Age 50–59	34.4 (26–44.6)	20.7 (15.4–27.6)	12.1 (9.1–15.9)	9.5 (7.1–12.5)	6.8 (5–9)	6.6 (4.9–9)
Age 60+	60.1 (47.3–73.3)	39.7 (30–51.3)	24.5 (18.5–31.9)	19.5 (14.6–25.7)	14.1 (10.5–18.9)	13.9 (10.2–19)
**IDU, No AIDS, Not virologically suppressed**						
Age 16–39	21.5 (15.6–29.3)	12.5 (8.9–17.5)	7.1 (5.1–9.9)	5.6 (3.9–7.8)	3.9 (2.8–5.6)	3.9 (2.7–5.6)
Age 40–49	32.3 (25.5–40.4)	19.3 (14.7–25.2)	11.2 (8.6–14.6)	8.8 (6.6–11.5)	6.3 (4.7–8.3)	6.2 (4.5–8.4)
Age 50–59	50.5 (40.2–61.8)	32.1 (24.4–41.5)	19.3 (14.7–25.2)	15.3 (11.4–20.3)	11 (8.1–14.9)	10.8 (7.8–14.9)
Age 60+	78.4 (66.2–88.6)	57 (44.7–70)	37.4 (28.7–47.8)	30.3 (22.8–39.6)	22.5 (16.5–30.2)	22.1 (15.9–30.3)
**IDU, AIDS, Virologically suppressed**						
Age 16–39	21.7 (15.4–29.9)	12.6 (8.8–17.7)	7.2 (5.1–10.1)	5.6 (3.9–7.9)	4 (2.8–5.6)	3.9 (2.7–5.6)
Age 40–49	32.5 (25.4–40.9)	19.4 (14.8–25.3)	11.3 (8.7–14.6)	8.8 (6.8–11.5)	6.3 (4.8–8.3)	6.2 (4.6–8.3)
Age 50–59	50.7 (40.3–62.2)	32.3 (24.7–41.5)	19.4 (14.9–25.2)	15.3 (11.6–20.1)	11.1 (8.3–14.7)	10.9 (8–14.8)
Age 60+	78.6 (66.5–88.7)	57.3 (45.4–69.7)	37.6 (29.2–47.4)	30.5 (23.3–39.2)	22.6 (16.9–29.8)	22.3 (16.3–29.9)
**IDU, AIDS, Not virologically suppressed**						
Age 16–39	33.4 (24.9–44)	20.1 (14.4–27.6)	11.7 (8.4–16.2)	9.1 (6.4–12.9)	6.5 (4.5–9.3)	6.4 (4.4–9.4)
Age 40–49	48 (39.4–57.5)	30.3 (23.5–38.4)	18.1 (14–23.3)	14.3 (10.8–18.7)	10.3 (7.7–13.7)	10.1 (7.4–13.8)
Age 50–59	69.3 (58.5–79.5)	47.8 (37.8–58.9)	30.3 (23.4–38.5)	24.3 (18.4–31.6)	17.8 (13.2–23.7)	17.5 (12.7–23.9)
Age 60+	92.4 (84.2–97.2)	75.8 (63.4–86.4)	54.4 (43.4–66.3)	45.4 (35.2–57.1)	34.7 (26–45.4)	34.3 (25.1–45.7)

*estimated from Weibull model

### Cause-specific mortality from ten years after starting ART

There were 218 deaths among 7,178 patients in the eight cohorts with ≥70% of cause of death information coded. It was possible to classify a cause of death in 180 (83%): the others were coded unknown/missing. The most frequent cause of death was non-AIDS non-liver cancer (41 deaths [25% of those classified]) followed by AIDS (35 [19%]), cardiovascular (22 [12%]) and liver-related (18 [10%]). Adjusted HRs for specific causes of death stratified by age, sex, CD4 count, viral suppression and AIDS are shown in Table 6. AIDS mortality was strongly related to lower CD4 count and viral replication at ten years after starting ART. Cardiovascular mortality was substantially higher in older patients (adjusted HR 8.95 [95% CI 3.63–22.1] comparing patients aged ≥60 with <60 years at ten years after ART start). Transmission via IDU and lower CD4 count at ten years after starting ART were strongly associated with mortality from liver-disease. Non-AIDS mortality was associated with both older age, adjusted HR compared with those aged <60 years 4.35 [95% CI 2.97–6.39], IDU status (3.40 [2.26–5.12]), lower CD4 (2.42 [1.49–3.94]) and prior AIDS diagnosis (1.58 [1.27–1.95]). Deaths from suicide/accident were higher in those with a prior AIDS diagnosis. Other causes of death (combining all causes with fewer than ten deaths) were strongly associated with older age (adjusted HR 5.71[2.84,11.5]).

**Table 6:** Adjusted hazard ratios (HR) for specific causes of death, adjusted for all variables in the table and stratified by cohort, deaths = 218.

		Adjusted HR (95% CI)					
Cause of death	N (%)	Age ≥60 vs. <60 years	Female vs. Male	IDU vs. non-IDU	CD4 <200 vs. ≥200 cells/μL	HIV-1 RNA >50 vs. ≤50 copies/mL	Prior AIDS diagnosis 10 years after ART start vs. no AIDS
**AIDS**	35 (16%)	1.08 (0.33, 3.58)	0.38 (0.13, 1.09)	1.24 (0.52, 2.96)	5.32 (2.49, 11.4)	4.90 (2.30, 10.4)	1.75 (1.16, 2.65)
**Non-AIDS**	145 (67%)	4.35 (2.97, 6.39)	0.70 (0.45, 1.06)	3.40 (2.26, 5.12)	2.42 (1.49, 3.94)	0.95 (0.62, 1.45)	1.58 (1.27, 1.95)
**Cardiovascular disease**	22 (10%)	8.95 (3.63, 22.1)	0.54 (0.16, 1.84)	2.20 (0.59, 8.23)	2.70 (0.71, 10.3)	1.30 (0.44, 3.80)	1.49 (0.84, 2.63)
**Liver related**	18 (8%)	1.65 (0.36, 7.50)	NA	3.84 (1.31, 11.2)	3.37 (1.11, 10.2)	2.37 (0.87, 6.40)	1.85 (1.02, 3.36)
**Non-AIDS, non-liver malignancies**	45 (21%)	3.39 (1.71, 6.70)	0.81 (0.39, 1.68)	1.79 (0.77, 4.20)	1.62 (0.55, 4.80)	0.58 (0.24, 1.42)	0.88, 1.97)
**Suicide/accident**	13 (6%)	2.55 (0.65, 10.0)	1.28 (0.39, 4.24)	2.45 (0.62, 9.69)	2.19 (0.42, 11.34)	0.65 (0.13, 3.17)	2.45 (1.25, 4.80)
Other*	47 (22%)	5.71 (2.84, 11.5)	0.85 (0.42, 1.73)	6.21 (3.14, 12.3)	2.57 (1.11, 5.95)	0.93 (0.44, 1.95)	1.52 (1.04, 2.21)
**Unknown**	38 (17%)	4.72 (2.28, 9.77)	0.50 (0.19, 1.27)	3.33 (1.48, 7.53)	3.38 (1.31, 8.74)	0.61 (0.24, 1.55)	1.15 (0.73, 1.83)

IDU: injection drug use, CI: confidence interval

* Causes with fewer than 10 deaths: these were non-AIDS Infection (8); heart or vascular (6); respiratory disease (5); chronic obstructive pulmonary disease (4); renal failure (4); substance abuse (4); digestive system (4); central nervous system (3); lung embolus (1); other causes not included in CoDe classification system (15)

## Discussion

### Summary

Among HIV positive patients who started combination ART 1996–1999 without prior exposure to antiretroviral drugs and survived for at least ten years, the majority were virally suppressed and had CD4 counts ≥500 cells/μL. Nonetheless lower CD4 count and lack of HIV-1 viral suppression at ten years after starting ART, and AIDS before or during the first decade of ART, were strong predictors of death during the second decade of ART. Low CD4 count at ART start was no longer associated with increased mortality beyond ten years after accounting for CD4 count measured at ten years after starting ART. Compared with patients from other risk groups, those who reported IDU as route of HIV transmission experienced higher mortality from ten years after ART start. The most frequently occurring causes of death were non-AIDS-defining malignancy, followed by AIDS, cardiovascular, and liver-related causes. Older age was strongly associated with cardiovascular mortality and with non-AIDS non-liver malignancies, IDU with non-AIDS infection and liver-related mortality, and low CD4 count and detectable viral replication at ten years after starting ART with AIDS mortality. The five-year mortality risk was <5% in 60% of patients and was strongly age-related: fewer than 30% of patients aged over 60 had five-year mortality risk less than 10%

### Strengths and limitations

Strengths of this study include its large sample size, which meant we could investigate at risk subgroups such as the IDU risk transmission group and older patients, and the inclusion of patients from geographically diverse clinical cohorts in Europe and North America. As with any observational study our results may have been affected by unmeasured confounders. We could only include a limited number of prognostic factors in our study. The VACS Index which includes hemoglobin, composite markers of liver and renal injury [FIB-4 and estimated glomerular filtration rate (eGFR)] and hepatitis C status is a better predictor of mortality than age, CD4 count and viral load alone, but many of the cohorts in ART-CC did not collect these data [22]. Our results may have been affected by exclusion of patients without CD4 count and viral load measured during the 10^th^ year of ART: such patients may attend care irregularly or may have been receiving care temporarily in a clinic not contributing data to ART-CC. Our results may only apply to patients who have survived 10 years on ART and continue to receive regular care. Outcomes are unknown in patients who were LTFU but CD4 counts and the proportion of patients virally suppressed were similar among those LTFU during the second decade of ART and those remaining alive and in care. Most cohorts link to death registries and so mortality rates among patients followed up in these cohorts should be reliably estimated[23], but deaths may have been under-ascertained in cohorts without links to death registries. Causes of death were classified according to a common protocol, but not all cohorts contributed data. However, the included deaths are likely representative of all deaths and we preferred not to increase the proportion of unknown or unclassifiable deaths by including cohorts with less complete data.

### Context of research

Even in this cohort of patients who have survived at least ten years on ART, traditional markers of HIV progression such as CD4 count, viral load, and AIDS events remain important risk factors warranting clinical attention. CD4 measurements at start of ART were unimportant compared to those at ten years which is consistent with previous findings that recent or current values of biomarkers best predict survival[13, 24] and that the prognostic importance of values measured at start of ART diminish with time[15, 25]. Indeed, we found that for patients with the same CD4 count at ten years after ART start, subsequent mortality was lower in patients whose CD4 count had risen more over the first decade of ART, which may represent a selection effect (“survival of the fittest”) as those who started ART with very low CD4 count were less likely to have survived 10 years and be included in this analysis. In this unselected treatment experienced population mortality rates remained substantially higher than in the general population. By contrast, standardised mortality rates in highly selected groups on long term ART who reached and maintained a CD4 count above 500 cells/ml have been found to approach that of the general population[26–28]. However, a recent study from Denmark found that even among well-treated HIV-infected individuals ≥50 years without comorbidity or AIDS-defining events the estimated median survival time remains lower than in the general population [29].

Older age was strongly associated with mortality, in particular that due to non-AIDS related morbidity. This suggests that provision of both preventive and therapeutic health care in older HIV-infected patients treated for many years will become increasingly important as the number of patients aged ≥60 years old increases. The most common cause of death was non-AIDS cancer, implying a need for preventive and screening measures adapted for use with HIV-positive patients who have survived long-term treatment with ART to be incorporated into their routine health care[30].

Only a fifth of classifiable causes of death were due to AIDS, consistent with previous studies that found low proportions of AIDS-related deaths in those treated for many years[26, 31, 32]. Although a substantial proportion of deaths soon after start of ART are AIDS-related,[5, 6, 8, 18, 24, 33–35] this proportion decreases with duration of ART. Correspondingly, the proportion (though not the rate), of non-AIDS related deaths increases with time on ART[26, 31, 32]. Consistent with our study, the Data Collection on Adverse events of Anti-HIV Drugs (D:A:D) study found that most frequent causes of death in the 2009–11 follow-up period were due to non-AIDS cancers, AIDS, cardiovascular disease, and liver disease[8].

It is only possible to study the long-term effects of antiretroviral therapy in patients who started ART many years ago. Patients in our study will have been treated with the less potent and more toxic drugs (compared with those currently available) available during 1996–99. Their management will have evolved as treatment guidelines changed and as new ART drugs and drug combinations were approved[2–4, 36]. Therefore long-term mortality of patients starting ART today can be expected to be lower than those found in our study. There is heterogeneity in mortality rates across cohorts[23] so that our estimates represent an average over included cohorts.

### Implications for health care

Given that a quarter of patients had detectable viral load at ten years after starting ART, careful monitoring of patients treated for many years remains essential. The high burden of cancer found in this and other HIV-infected populations[37] indicates the need to optimise and improve adherence to screening guidelines[38, 39]. Higher mortality among patients diagnosed with AIDS before ART start or within the first decade of treatment may arise from chronic or late-onset sequelae of AIDS-defining illnesses, including relapse, secondary malignancy, heart disease after anti-cancer chemotherapy[40] or chronic lung injury following infection with *Mycobacterium tuberculosis*[41] or *Pneumocystis jirovecii*[42].

Patients reporting HIV transmission via IDU remained at considerably higher risk of mortality, particularly death from liver-disease, beyond ten years after starting ART. This higher mortality could arise from continued drug-use, co-infection with hepatitis C virus, higher rates of smoking and alcohol abuse, or poorer adherence to treatment[43, 44]. Several participating cohorts only assess IDU at enrolment, therefore we could not differentiate whether transmission risk of IDU remained a risk factor because of persisting drug use or because of direct sequelae or adverse socioeconomic factors associated with historic drug use. It is likely that the association of ongoing drug use with deaths from non-AIDS infection and substance use is much greater than that estimated for the transmission risk group designated IDU in this study. Current, but not former, drug use has been found to be associated with lack of adherence to ART which in turn correlates with virological outcomes[45]. It is therefore important to address continuing misuse of drugs and alcohol which may require opium substitution therapy or counselling. IDU should be screened for hepatitis C and offered treatment if warranted. Smokers should be offered nicotine substitution therapy and smoking cessation programs. Reducing mortality in IDU may also require interventions to address depression and social deprivation.

### Conclusion

This study of patients who have survived ten years after starting ART found that CD4 count and viral load remain important for prognosis. However, deaths were mostly non-AIDS-related, and care for these patients should focus on improving management of non-HIV morbidity, in particular risk factors and screening for non-AIDS cancer. Whilst the majority of patients have low 5-year mortality risk, older patients have much higher risk. Patients with presumed transmission via IDU also have worse prognosis and likely require intensive management with a variety of interventions that aim to reduce their excess mortality. Our estimates of prognosis beyond ten years of ART could inform mathematical models[46] used to predict outcomes and allow comparisons to be made against the observed data to test the validity of such models. Our findings could also inform estimates of the future cost of treatment of people living with HIV.

## Appendix 1

Cohorts included in this paper were the French Hospital Database on HIV (FHDH); the Italian Cohort of Antiretroviral-naïve patients (ICONA); the Swiss HIV Cohort Study (SHCS); the AIDS Therapy Evaluation project, Netherlands (ATHENA); The Multicenter Study Group on EuroSIDA; the Aquitaine Cohort; the Royal Free Hospital Cohort, UK; the South Alberta Clinic Cohort; Cohorte de la Red de Investigación en Sida (CoRIS), Spain; The Danish HIV Cohort Study, Denmark; HAART Observational Medical Evaluation and Research (HOMER), Canada; HIV Atlanta Veterans Affairs Cohort Study (HAVACS), USA; Osterreichische HIV-Kohortenstudie (OEHIVKOS), Austria; Proyecto para la Informatizacion del Seguimiento Clinico-epidemiologico de la Infeccion por HIV y SIDA (PISCIS), Spain; VACH, Spain; Veterans Aging Cohort Study (VACS), USA; Vanderbilt, USA; University of Washington HIV Cohort, USA; and the Koln/Bonn Cohort.

## References

[1] Bartlett JA, DeMasi R, Quinn J, Moxham C, Rousseau F. Overview of the effectiveness of triple combination therapy in antiretroviral-naive HIV-1 infected adults. AIDS. 2001;15(11):1369–77. 11504958 10.1097/00002030-200107270-00006

[2] Blanco JL, Whitlock G, Milinkovic A, Moyle G. HIV integrase inhibitors: a new era in the treatment of HIV. Expert opinion on pharmacotherapy. 2015;16(9):1313–24. 10.1517/14656566.2015.1044436 26001181

[3] Shubber Z, Calmy A, Andrieux-Meyer I, Vitoria M, Renaud-Thery F, Shaffer N, et al Adverse events associated with nevirapine and efavirenz-based first-line antiretroviral therapy: a systematic review and meta-analysis. AIDS. 2013;27(9):1403–12. 10.1097/QAD.0b013e32835f1db0 23343913

[4] Astuti N, Maggiolo F. Single-Tablet Regimens in HIV Therapy. Infectious diseases and therapy. 2014;3(1):1–17. 10.1007/s40121-014-0024-z 25134808 PMC4108118

[5] The Antiretroviral Therapy Cohort C. Causes of death in HIV-1 infected patients treated with antiretroviral therapy 1996–2006: collaborative analysis of 13 HIV cohort studies. Clin Infect Dis. 2010;50(10):1387–96. 10.1086/652283 20380565 PMC3157754

[6] Ingle SM, May MT, Gill MJ, Mugavero MJ, Lewden C, Abgrall S, et al Impact of risk factors for specific causes of death in the first and subsequent years of antiretroviral therapy among HIV-infected patients. Clin Infect Dis. 2014;59(2):287–97. 10.1093/cid/ciu261 24771333 PMC4073781

[7] Lima V, Lourenço L, Yip B, Hogg B, Phillips P, Montaner J. Trends in AIDS incidence and AIDS-related mortality in British Columbia between 1981 and 2013. Lancet HIV. 2015;2(3):92–7. 10.1016/S2352-3018(15)00017-X PMC4357843 25780802

[8] Smith CJ, Ryom L, Weber R, Morlat P, Pradier C, Reiss P, et al Trends in underlying causes of death in people with HIV from 1999 to 2011 (D:A:D): a multicohort collaboration. Lancet. 2014;384(9939):241–8. 10.1016/S0140-6736(14)60604-8 25042234

[9] Egger M, May M, Chene G, Phillips AN, Ledergerber B, Dabis F, et al Prognosis of HIV-1-infected patients starting highly active antiretroviral therapy: a collaborative analysis of prospective studies. Lancet. 2002;360(9327):119–29. 12126821 10.1016/s0140-6736(02)09411-4

[10] May M, Sterne JA, Sabin C, Costagliola D, Justice AC, Thiebaut R, et al Prognosis of HIV-1-infected patients up to 5 years after initiation of HAART: collaborative analysis of prospective studies. AIDS. 2007;21(9):1185–97. 17502729 10.1097/QAD.0b013e328133f285 PMC3460385

[11] Kowalska JD, Friis-Moller N, Kirk O, Bannister W, Mocroft A, Sabin C, et al The Coding Causes of Death in HIV (CoDe) Project: initial results and evaluation of methodology. Epidemiology. 2011;22(4):516–23. 10.1097/EDE.0b013e31821b5332 21522013

[12] Ehren K, Hertenstein C, Kummerle T, Vehreschild JJ, Fischer J, Gillor D, et al Causes of death in HIV-infected patients from the Cologne-Bonn cohort. Infection. 2014;42(1):135–40. 10.1007/s15010-013-0535-7 24081925

[13] Lewden C, Bouteloup V, De Wit S, Sabin C, Mocroft A, Wasmuth JC, et al All-cause mortality in treated HIV-infected adults with CD4 >/ = 500/mm3 compared with the general population: evidence from a large European observational cohort collaboration. International journal of epidemiology. 2012;41(2):433–45. 10.1093/ije/dyr164 22493325

[14] Obel N, Omland LH, Kronborg G, Larsen CS, Pedersen C, Pedersen G, et al Impact of non-HIV and HIV risk factors on survival in HIV-infected patients on HAART: a population-based nationwide cohort study. PLoS One. 2011;6(7):e22698 10.1371/journal.pone.0022698 21799935 PMC3143183

[15] Lanoy E, May M, Mocroft A, Phillip A, Justice A, Chene G, et al Prognosis of patients treated with cART from 36 months after initiation, according to current and previous CD4 cell count and plasma HIV-1 RNA measurements. AIDS. 2009;23(16):2199–208. 10.1097/QAD.0b013e3283305a00 19779320 PMC3122149

[16] May MT, Ingle SM, Costagliola D, Justice AC, de Wolf F, Cavassini M. Cohort profile: Antiretroviral Therapy Cohort Collaboration (ART-CC). IJE. 2013:12. 10.1093/ije/dyt010 PMC4052127 23599235

[17] Kowalska JD, Kirk O, Mocroft A, Hoj L, Friis-Moller N, Reiss P, et al Implementing the number needed to harm in clinical practice: risk of myocardial infarction in HIV-1-infected patients treated with abacavir. HIV Med. 2010;11(3):200–8. 10.1111/j.1468-1293.2009.00763.x 19863618

[18] Lewden C, May T, Rosenthal E, Burty C, Bonnet F, Costagliola D, et al Changes in causes of death among adults infected by HIV between 2000 and 2005: The "Mortalite 2000 and 2005" surveys (ANRS EN19 and Mortavic). Journal of Acquired Immune Deficiency Syndromes. 2008;48(5):590–8. 10.1097/QAI.0b013e31817efb54 18645512

[19] May M, Porter K, Sterne JA, Royston P, Egger M. Prognostic model for HIV-1 disease progression in patients starting antiretroviral therapy was validated using independent data. Journal of clinical epidemiology. 2005;58(10):1033–41. 16168349 10.1016/j.jclinepi.2005.02.015

[20] Royston P, Parmar MK. Flexible parametric proportional-hazards and proportional-odds models for censored survival data, with application to prognostic modelling and estimation of treatment effects. Stat Med. 2002;21(15):2175–97. 12210632 10.1002/sim.1203

[21] Fine J, Gray R. A Proportional Hazards Model for the Subdistribution of a Competing Risk. Journal of the American Statistical Association. 1999;94(446):496–509.

[22] Tate JP, Justice AC, Hughes MD, Bonnet F, Reiss P, Mocroft A, et al An internationally generalizable risk index for mortality after one year of antiretroviral therapy. AIDS. 2013;27(4):563–72. 10.1097/QAD.0b013e32835b8c7f 23095314 PMC4283204

[23] May MT, Hogg RS, Justice AC, Shepherd BE, Costagliola D, Ledergerber B, et al Heterogeneity in outcomes of treated HIV-positive patients in Europe and North America: relation with patient and cohort characteristics. International journal of epidemiology. 2012;41(6):1807–20. 10.1093/ije/dys164 23148105 PMC3535877

[24] Leone S, Gregis G, Quinzan G, Velenti D, Cologni G, Soavi L, et al Causes of death and risk factors among HIV-infected persons in the HAART era: analysis of a large urban cohort. Infection. 2011;39(1):13–20. 10.1007/s15010-010-0079-z 21246246

[25] Young J, Psichogiou M, Meyer L, Ayayi S, Grabar S, Raffi F, et al CD4 cell count and the risk of AIDS or death in HIV-Infected adults on combination antiretroviral therapy with a suppressed viral load: a longitudinal cohort study from COHERE. PLoS medicine. 2012;9(3):e1001194 10.1371/journal.pmed.1001194 22448150 PMC3308938

[26] Lewden C, Chene G, Morlat P, Raffi F, Dupon M, Dellamonica P, et al HIV-infected adults with a CD4 cell count greater than 500 cells/mm3 on long-term combination antiretroviral therapy reach same mortality rates as the general population. J Acquir Immune Defic Syndr. 2007;46(1):72–7. 17621240 10.1097/QAI.0b013e318134257a

[27] McManus H, O'Connor CC, Boyd M, Broom J, Russell D, Watson K, et al Long-term survival in HIV positive patients with up to 15 Years of antiretroviral therapy. PLoS One. 2012;7(11):e48839 10.1371/journal.pone.0048839 23144991 PMC3492258

[28] May MT, Gompels M, Delpech V, Porter K, Orkin C, Kegg S, et al Impact on life expectancy of HIV-1 positive individuals of CD4+ cell count and viral load response to antiretroviral therapy. AIDS. 2014;28(8):1193–202. 10.1097/QAD.0000000000000243 24556869 PMC4004637

[29] Legarth RA, Ahlstrom MG, Kronborg G, Larsen CS, Pedersen C, Pedersen G, et al Long-Term Mortality in HIV-Infected Individuals 50 Years or Older: A Nationwide, Population-Based Cohort Study. J Acquir Immune Defic Syndr. 2016;71(2):213–8. 10.1097/QAI.0000000000000825 26334734

[30] Sigel K, Dubrow R, Silverberg M, Crothers K, Braithwaite S, Justice A. Cancer screening in patients infected with HIV. Current HIV/AIDS reports. 2011;8(3):142–52. 10.1007/s11904-011-0085-5 21695529 PMC3307131

[31] Palella FJ Jr., Baker RK, Moorman AC, Chmiel JS, Wood KC, Brooks JT, et al Mortality in the highly active antiretroviral therapy era: changing causes of death and disease in the HIV outpatient study. J Acquir Immune Defic Syndr. 2006;43(1):27–34. 16878047 10.1097/01.qai.0000233310.90484.16

[32] Kowalska JD, Reekie J, Mocroft A, Reiss P, Ledergerber B, Gatell J, et al Long-term exposure to combination antiretroviral therapy and risk of death from specific causes: no evidence for any previously unidentified increased risk due to antiretroviral therapy. AIDS. 2012;26(3):315–23. 10.1097/QAD.0b013e32834e8805 22112597

[33] Kowalska JD, Mocroft A, Ledergerber B, Florence E, Ristola M, Begovac J, et al A standardized algorithm for determining the underlying cause of death in HIV infection as AIDS or non-AIDS related: results from the EuroSIDA study. HIV clinical trials. 2011;12(2):109–17. 10.1310/hct1202-109 21498154

[34] Monforte A, Abrams D, Pradier C, Weber R, Reiss P, Bonnet F, et al HIV-induced immunodeficiency and mortality from AIDS-defining and non-AIDS-defining malignancies. AIDS. 2008;22(16):2143–53. 10.1097/QAD.0b013e3283112b77 18832878 PMC2715844

[35] Data Collection on Adverse Events of Anti HIVdSG, Smith C, Sabin CA, Lundgren JD, Thiebaut R, Weber R, et al Factors associated with specific causes of death amongst HIV-positive individuals in the D:A:D Study. AIDS. 2010;24(10):1537–48. 10.1097/QAD.0b013e32833a0918 20453631

[36] Mocroft A, Reiss P, Rakhmanova A, Banhegyi D, Phillips AN, De Wit S, et al A survey of ATRIPLA use in clinical practice as first-line therapy in HIV-positive persons in Europe. Infection. 2014;42(4):757–62. 10.1007/s15010-014-0630-4 24902520 PMC4103996

[37] Wells JS, Holstad MM, Thomas T, Bruner DW. An integrative review of guidelines for anal cancer screening in HIV-infected persons. AIDS patient care and STDs. 2014;28(7):350–7. 10.1089/apc.2013.0358 24936878

[38] Laut KG, Mocroft A, Lazarus J, Reiss P, Rockstroh J, Karpov I, et al Regional differences in self-reported HIV care and management in the EuroSIDA study. J Int AIDS Soc. 2014;17(4 Suppl 3):19504 10.7448/IAS.17.4.19504 25394013 PMC4224929

[39] Thorsteinsson K, Ladelund S, Jensen-Fangel S, Katzenstein TL, Johansen IS, Pedersen G, et al Adherence to the cervical cancer screening program in women living with HIV in Denmark: comparison with the general population. BMC Infect Dis. 2014;14:256 10.1186/1471-2334-14-256 24885577 PMC4025560

[40] Siegel R, DeSantis C, Virgo K, Stein K, Mariotto A, Smith T, et al Cancer treatment and survivorship statistics, 2012. CA: a cancer journal for clinicians. 2012;62(4):220–41. 22700443 10.3322/caac.21149

[41] Kim HW, Song KS, Goo JM, Lee JS, Lee KS, Lim TH. Thoracic Sequelae and Complications of Tuberculosis. RadioGraphics. 2001;21:839–60. 11452057 10.1148/radiographics.21.4.g01jl06839

[42] Swain SD, Han S, Harmsen A, Shampeny K, Harmsen AG. Pulmonary hypertension can be a sequela of prior Pneumocystis pneumonia. The American journal of pathology. 2007;171(3):790–9. 17640969 10.2353/ajpath.2007.070178 PMC1959506

[43] May MT, Justice AC, Birnie K, Ingle SM, Smit C, Smith C, et al Injection Drug Use and Hepatitis C as Risk Factors for Mortality in HIV-Infected Individuals: The Antiretroviral Therapy Cohort Collaboration. J Acquir Immune Defic Syndr. 2015;69(3):348–54. 10.1097/QAI.0000000000000603 25848927 PMC4506784

[44] Weber R, Huber M, Battegay M, Stahelin C, Castro Batanjer E, Calmy A, et al Influence of noninjecting and injecting drug use on mortality, retention in the cohort, and antiretroviral therapy, in participants in the Swiss HIV Cohort Study. HIV Med. 2015;16(3):137–51. 10.1111/hiv.12184 25124393

[45] Weber R, Huber M, Rickenbach M, Furrer H, Elzi L, Hirschel B, et al Uptake of and virological response to antiretroviral therapy among HIV-infected former and current injecting drug users and persons in an opiate substitution treatment programme: the Swiss HIV Cohort Study. HIV Med. 2009;10(7):407–16. 10.1111/j.1468-1293.2009.00701.x 19490174

[46] Phillips AN, Cambiano V, Nakagawa F, Brown AE, Lampe F, Rodger A, et al Increased HIV incidence in men who have sex with men despite high levels of ART-induced viral suppression: analysis of an extensively documented epidemic. PLoS One. 2013;8(2):e55312 10.1371/journal.pone.0055312 23457467 PMC3574102

